# Abstract of Proceedings of American Medical Association

**Published:** 1852-06

**Authors:** 


From the New Jersey Med. Reporter.

*Abstract of Proceedings of the American Medical Association held at Richmond, Va.*, May 4, 1852.

The Association met in the Second Presbyterian Church, at 11 o'clock. The President, Dr. Moultrie, of Charleston, in the chair.

Dr. James Beale, Chairman of the Committee of Reception, welcomed the delegates to the city of Richmond.

Dr. Haxall, Chairman of Committee of Arrangements, read a list of the delegates who were present, and registered.

Dr. Hays, of Pa., offered the following resolution:

*Resolved*, That a committee of one from each State, to be selected by its delegation, be appointed to nominate suitable officers for the Association.

The resolution having been adopted, a recess of ten minutes was taken, to allow the delegations opportunity to appoint the nominating committee.

At the expiration of the recess, the President announced the following committee:

NOMINATING COMMITTEE.

| | | | |
|---|---|---|---|
| Isaac Lincoln, | Maine, | J. H. Dickson, | N. C. |
| Jeremiah Blake, | N. H. | H. R. Frost, | S. C. |
| Jacob Bigelow, | Mass. | C. B. Nottingham, | Geo. |
| H. W. Rivers, | R. I. | A. Lopez, | Ala. |
| Chas. Hooker, | Conn. | W. L. Sutton, | Ky. |
| Jos. M. Smith, | N. Y. | C. A. Pope, | Mo. |
| G. R. Chetwood, | N. J. | D. Tilden, | Ohio. |
| G. W. Norris, | Penn. | D. Brainard, | Ill. |
| H. T. Askew, | Del. | Z. Pitcher, | Mich. |
| G. S. Gibson, | Md. | J. H. Rauch, | Iowa. |
| C. Boyle, | D. C. | Paul T. Eve, | Tenn. |

James Beale, Va.

After the calling of the roll, the President delivered the annual address.

The nominating committee then reported the following gentleman officers of the Association for the present year:

*For President*—BEVERLY R. WELLFORD, M. D., of Va.

*For Vice Presidents*—JONATHAN KNIGHT, M. D., of Conn., JAMES W. THOMPSON, M. D., of Del., THOS. S. SIMONS, M. D., of S. C., CHAS. A. POPE, M. D., Missouri.

*For Treasurer*—D. FRANCIS CONDIE, M. D., Pa.

The officers thus nominated were duly elected, and the committee requested to nominate Secretaries and decide upon the next place of meeting.

On motion, Drs. John L. Atlee, of Penn., Haxall, of Va., and Eve, of Tenn., were appointed a committee to wait upon Dr. Wellford, and announce his election, and conduct him to the chair. Dr. Wellford having taken the chair returned his thanks for the honor conferred upon him.

Dr. Haxall, of Va., offered the following preamble and resolution, which were unanimously adopted:

"The American Med. Society in Paris being so constituted, that it would be entitled to representation if it existed in this country, and as it is recognized abroad as an American institution;

"*Resolved*, That the delegates accredited to the Association by the American Medical Society in Paris, be, and are hereby invited to take seats in this body."

Dr. Paul Lajus offered the following resolution, which was adopted:

*Resolved*, That Dr. Brown-Sequard, of Paris be invited to occupy a seat among the delegates at the present meeting of the Association.

Dr. Isaac Hays read the report of the committee on publication, and the report of the Treasurer. They were received, and the following resolutions appended to the report of the committee of publication were unanimously adopted:

*Resolved*, That the assessment for the present year be three dollars.

*Resolved*, That the committee of publication be authorized to fix the price at which the transactions of the present year will be furnished to such of the members of the Association as shall remit the amount decided upon by the committee within a specified time (to be fixed by them). And that it shall be the duty of the said committee, to issue a circular, informing the members of the terms upon which the transactions will be furnished them.

*Resolved*, That the committee be further authorized to take such measures in the disposal of the copies of the Transactions, remaining after all such numbers are supplied, as shall comply with the terms set forth in the circular of the committee, as they may deem expedient.

Dr. Hayward presented the report from the committee on prize essays, and broke the seal of the packet, containing the name of the author of the essay entitled, "On Variations of Pitch in Percussion and Respiratory Sounds, and their Application to Physical Diagnosis," which was deemed worthy of the prize. The author proved to be Dr. Austin Flint, of Buffalo, N. Y., to whom the prize was awarded, and the report referred to the committee of publication.

The report of the committee on the medical botany of the U. S. for 1850–51 from Dr. A. Clapp, Conn., was presented and referred to the committee of publication.

Dr. R. W. Haxall, of Va., read a short report of the progress of the Committee on the epidemics of Virginia and North Carolina, and asked to be continued; which request was granted.

On motion of Dr. Gooch, the editorial corps were invited to take seats on the floor.

The Secretary stated that he had transmitted copies of the preamble and resolutions of 1850–51, relative to the assimilated rank of the medical staff of the army and navy, to the several departments ordered by the resolutions, and that the chief of the Bureau of Medicine and Surgery at Washington had approved the course recommended by the Association in a letter which was read. Dr. Pinkney, of the Navy, asked leave to read a memorial, which he had prepared to present to Congress on this subject, which he sustained in a lengthened and forcible speech—after which Dr. Cox, of Md., offered resolutions expressive of the approval of the Association in the matter, &c., &c.; which resolutions were referred to a special committee.

Dr. Simons, of S. C., offered the following resolutions:

*Resolved*, That the American Medical Association do memorialize Congress to require all vessels bringing steerage passengers over to have a surgeon on board.

*Resolved*, That a committee of this Association be appointed to draw up a memorial to Congress, making such suggestions as they may deem fit as regards the importance of this measure.

The report of the committee upon the constitution being the special order, Dr. Hays, chairman of the committee, made a report. Dr. T. H. Yardley, a member of the committee, presented a counter-report. Much discussion ensued, and many resolutions and amendments were proposed, and withdrawn in favor of the following resolution, offered by Dr. Thomas, of Md., and amended by Dr. Stewart, of N. Y.

*Resolved*, That the two reports, on proposed alterations of the Constitution, be referred to a committee of three, to be appointed by the chair, with instructions to report to-morrow morning in definite and proper form, such amendments as will embrace the views set forth in the reports, and such other views as may appear to them advisable.

The resolution was adopted.

The Secretary read the following communication from the New York Academy of Medicine, which on motion was referred to the publishing committee, and ordered to be printed:

NEW YORK ACADEMY OF MEDICINE,
NEW YORK, April 22, 1852.

Sir: I have the honor to transmit to you a copy of the preamble and resolutions adopted at a regular meeting of the New York Academy of Medicine, held April 21, 1852:

Whereas, The clinics, now held at the medical colleges, as at present conducted, are, or may be made tributary to the private interests of the professors, at the expense of other and younger members of the profession: depriving them, by an odious monopoly, of practice and operations, and often of *fees* to which they are justly entitled,

Therefore *Resolved*, As the sense of this Academy, that to prescribe or practice upon the legitimate patients of any other physician, knowing them to be such, although done gratuitously at a clinic, is equally unwarrantable and unprofessional, with similar interference with the patients of another in private practice, and in either case is a violation of the code of medical ethics adopted by this body.

*Resolved*, That the possible perversion of these clinics, to the private emolument of those conducting them, by transferring patients to their private offices, and then exacting fees from those found able to pay, divests the clinics of all pretext for professing to be public charities, and should be scrupulously guarded against in all our colleges by stringent rules.

*Resolved*, That a copy of these resolutions be sent to the authorities of the several medical colleges in this city.

The Secretary was also instructed to forward a copy of these resolutions to the American Medical Association.

Respectfully yours,
JACKSON BALTON, M.D., *Rec. Sec.*

Dr. Eve, from the committee on nominations, recommended the following additional officers for the ensuing year:

*For Secretaries*—Drs. P. CLAIBORNE GOOCH, of Va., and ED. L. BEADLE, of N. Y.

*Committee of Publication*—Drs. D. F. Condie, of Pa., Isaac Hays, of Pa., P. C. Gooch, of Va., E. L. Beadle, of N. Y., Isaac Parrish, of Pa., G. Emerson, of Pa., G. W. Norris, of Pa.

*Committee of Arrangements*—Drs. T. Campbell Stewart, of N. Y., John Watson, of N. Y., Wm. Rockwell, of N. Y., James R. Wood, of N. Y., Robt. Watts, Jr., of N. Y., Alfred Post, of N. Y., John G. Adams, of N. Y., H. D. Bulkley, of N. Y.

On motion, the report was received, and the gentlemen named were unanimously elected officers of the Association.

The report of the committee on "The Blending and Conversion of the Types of Fever," was next read by Dr. C. B. Williman, S. C. (in place of Dr. Dickson, not present), and referred to the committee of Publication.

Dr. Hayward, of Mass., presented and read a report upon the Radical Cure of Hernia, which was also referred to the publishing committee.

Dr. Greene, of N. Y., offered the following resolutions:

*Resolved*, That at all future meetings of this Association, the reports of committees and all contributions on scientific subjects, occupying more than ten pages of quarto post manuscript, be accompanied each by an abstract or synopsis embracing the principal points of such report or paper, which abstract or synopsis may be read before the Association.

*Resolved*, That the above resolution be transmitted to the chairman of each committee.

The thanks of the Association were presented to the physicians and citizens of Virginia, to the Trustees of the Second Presbyterian Church, to the Committee of Arrangements, and to the Managers of the Danville Railroad Company for their kindness and attention to its members.

The following committee was appointed to report what action this Association should take in order to procure, for distribution among the medical profession of the United States, a large edition of the medical statistics of the country, as furnished by the late census, viz.: Drs. Simons, of S. C., Boyle, of D. C., Sumner, of Conn.

The following committee was appointed to report on the relation between climate and pulmonary consumption, viz.: Drs. D. F. Condie, R. E. Rogers, James M. Smith, Moultrie, and McGuire.

On motion of Dr. Rockwell, it was *Resolved*, That the Committee to memorialize Congress on the subject of compelling passenger vessels to carry surgeons, be directed also to call their attention to the importance of giving to each steerage passenger a certain amount of space between decks.

The nominating committee reported the following special committees:

D. F. Condie, Philadelphia, Causes of Tubercular Disease.

James Jones, New Orleans, The Mutual Relations of Yellow and Bilious Remittent Fevers.

R. S. Homes, St. Louis, Missouri, Epidemic Erysipelas.

Charles D. Meigs, Philadelphia, Acute and Chronic Diseases of the neck of the Uterus.

J. P. Jervey, Charleston, South Carolina, Dengue.

Daniel Drake, Cincinnati, Milk Sickness, so called.

Dr. Lopez, Mobile, Prevalence of Idiopathic Tetanus,

George B. Wood, Philadelphia, Diseases of Parasitic Origin.

R. D. Arnold, Savannah, the Physiological Peculiarities and Diseases of Negroes.

Joseph Carson, Philadelphia, Alkaloids which may be substituted for Quinia.

S. D. Gross, Louisville, Ky., Results of Surgical operations for the relief of Malignant Diseases.

James R. Wood, New York, Statistics of the operation for the removal of Stone in the Bladder.

Alexander H. Stevens, New York, Sanitary Principles applicable to the Construction of Dwellings.

G. Emerson, Philadelphia, Agency of the Refrigeration produced through Upward Radiation of Heat, as an Exciting Cause of Disease.

Henry J. Bigelow, Boston, The best Means of making Pressure in Reducible Hernia.

A. T. B. Merritt, Richmond, Cholera and its Relations to Congestive Fever; their Analogy or Identity.

Usher Parsons, Providence, R. I., Displacement of the Uterus.

H. F. Campbell, Augusta, Ga., Typhoid Fever.

Worthington Hooker, Connecticut, Epidemics of New England and New York.

Jno. L. Atlee, Lancaster, Pa., Epidemics of New Jersey, Pennsylvania, Delaware, and Maryland.

Robt. W. Haxall, Richmond, Va., Epidemics of Virginia and North Carolina.

William M. Boling, Montgomery, Ala., Epidemics of South Carolina, Georgia, Florida, and Alabama.

Edward H. Barton, New Orleans, Epidemics of Mississippi, Louisiana, Texas, and Arkansas.

Dr. Sutton, Georgetown, Ky., Epidemics of Tennessee and Kentucky.

Thomas Reyburn, St. Louis, Mo., Epidemics of Missouri, Illinois, Iowa, and Wisconsin.

George Mendenhall, Cincinnati, Epidemics of Ohio, Indiana, and Michigan.

*Committee on Volunteer Communications*—Joseph M. Smith, Jno. A Swett, Willard Parker, Gurdon Buck, Alfred C. Post, all of New York.

The titles of the reports on epidemics and other special reports were read, and they were referred to the committee on publication.

The subject of altering several important provisions of the Constitution having been presented in a majority and minority report, both submitted by different portions of a committee, to whom the subject was referred last year, claimed a considerable share of attention in the Association, and much interesting discussion ensued in which some of the most distinguished men of our profession participated; and the Committee who were charged with condensing the subject of both reports into one, so far as they could be made compatible with each other, presented a series of well-digested propositions, which, after free discussion and some alterations, were laid on the table till next year, then to be taken up and finally acted upon. These propositions embrace the absorbing question of delegation, and present a conservative policy, which may probably be adopted, as it is evident the Association is not yet fully prepared to exclude the schools from a representation at its meetings.

The committee suggests that delegates shall be appointed for one year; that they shall hold their appointment from County and State Medical Societies, from chartered medical colleges, hospitals, and permanent voluntary medical associations in good standing with the profession, and from the medical staffs of the army and navy. The county, district, and voluntary chartered medical societies, shall have the privilege of sending one delegate for every ten of its resident members, and one more for every additional fraction of more than one-half that number; while every State Society may send four delegates, and, in those States in which county and district societies are not generally organized, they shall be entitled to send one for every ten regular members, and one more for every additional fraction of more than half of this number. No medical society shall have the privilege of representation which does not require of its members an observance of the code of ethics adopted by the Association. Every chartered medical college, acknowledging fealty to the code of ethics, having six professors, and giving a course of lectures of not less than sixteen weeks annually, requiring also of students three years study; and hospitals, and lunatic asylums, having accommodations for one hundred patients, shall be entitled each to one delegate. The University of Virginia, in consequence of its peculiar mode of instruction and examination, was mentioned as worthy of representation, notwithstanding it does not comply, in some respects, with the terms of the proposition; but the exception in its favor is only to be continued so long as the present system of teaching shall remain in force.

